# Recent trends for the management of diabetes for older adults in the context of universal coverage and COVID-19: evidence from Mexico

**DOI:** 10.1093/inthealth/ihaa098

**Published:** 2020-11-18

**Authors:** Armando Arredondo

**Affiliations:** National Institute of Public Health. Av Universidad 655, Col Sta Maria, Cuernavaca, Mexico

**Keywords:** diabetes management, economic burden, epidemiological trends, older adults

## Abstract

**Background:**

To identify trends in the epidemiological and economic burden of diabetes in the elderly.

**Methods:**

Using the Box–Jenkins method to estimate expected cases for the period 2020–2022, costs were determined with the instrumentation technique. The population base was 4 032 189 older adults diagnosed with diabetes in 2019.

**Results:**

Regarding the epidemiological burden, there is an increase of 11–15% (p<0.001). Comparing the economic burden for 2020 vs 2022, the increase is estimated as 29% (p<0.05).

**Conclusion:**

In the framework of the coronavirus disease 2019 pandemic, the increase in diabetes cases and costs in older adults substantially complicates the scope of universal coverage for patients with diabetes.

## Introduction

Within the framework of the United Nations 2030 agenda,^1^ the strategy of universal health coverage for non-communicable diseases in older adults represents one of the main problems to be solved in Latin America. In this context, diabetes care, due to its growth in recent years, is one of the main health challenges for older adults in the short, medium and long term. Indeed, some projections for diabetes in older adults predict that by 2030 cases will increase by 207% for middle-income countries and 81% for high-income countries.^2^ Correspondingly, the global economic burden from 2017–2045 is projected to increase dramatically for diabetes and comorbid conditions. The high costs of healthcare and changes in demographics, in which a change in the population pyramid is seen, will add risks for the adult population and increase the demand for health services in the near future.^2^

On the other hand, in the framework of the coronavirus disease 2019 (COVID-19) pandemic, the epidemiological and economic challenges are more complicated, mainly because it is precisely in older adults with diabetes that the effects of COVID-19 are more intense and lethal.^3^ It is important to highlight that regarding the COVID-19 pandemic in Mexico, as of 14 October 2020 there were 829 396 confirmed cases and in 38% (315 134 cases) there was comorbidity associated with diabetes or hypertension, with 84 898 deaths reported.^3^

As part of the history of universal coverage, the most recent reform of the Mexican health system was proposed in 2002 and implementation began in 2003.^4^ The complexity in the design and implementation of the universal coverage strategy for diabetes requires some clarifications. The universal coverage scheme for health services is operated by the Ministry of Health through a package of services that guarantees financial protection for 40% of the population, approximately 48 000 000 Mexicans (50% of the population is covered by social security institutions and 10% by the private sector). In the case of diabetes, as part of the national strategy in response to diabetes in older adults (slightly more than 4 million patients in 2019^5^^–^^6^), this stage included the implementation of a package of basic healthcare services, laboratory studies and drugs that should be provided and guarantees a reduction in health-related out-of-pocket expenses.^4^

In this context, the purpose of this brief report is to present recent trends expected for the period 2020–2022 regarding on the epidemiological and economic burden for diabetes in older adults, highlighting the implications and challenges in terms of universal coverage in the framework of the COVID-19 pandemic.

## Methods

Using forecasting models, the findings presented herein come from an update of a cost estimation model and financial consequences of epidemiological changes. The new evidence is part of a 3-y (2020–2022) data update strategy. Data in the forecasting model come from a macro-longitudinal study of epidemiological changes in Mexico due to diabetes.^5^ The population base consisted of 4 032 189 cases of older adults diagnosed with diabetes in 2019.^6^ The healthcare institutions under study belong to the public health sector in Mexico. The research protocol and letter of informed consent were reviewed and approved by the ethics committee of the National Institute of Public Health of Mexico.

Starting with the Box–Jenkins method,^7^ autoregressive integrated moving average models were used, based on the coefficient of partial autocorrelation. For the time series, data from annual cases diagnosed and under diabetes control were used for the period 1996–1919 in the main institutions providing health services in Mexico. Regarding the cost methodology, direct costs refers to the average cost for annual case management per patient with the five main complications. Healthcare services were obtained from the management of standardized cases, adjusted by type of institution. The standardization and adjustment for the type of institution was performed with the application of a discount rate of 2% annually, based on the cost of annual average case handling and cost of inputs by type of institution. Direct costs did not include transportation expenses or lost productivity due to waiting times. The indirect costs were determined using the human capital model developed for chronic diseases in Latin America.^8^ A probabilistic model was designed to include three categories of monetary costs attributable to diabetes in three public institutions: mortality costs, costs of permanently disabled patients and costs of temporarily disabled patients.

It is important to note that since this is a short report of results on an update of recent trends in the prognosis of diabetes cases in older adults, the methods of the model are not given in detail, nor are the cost estimates. The methodological details have been published previously in other manuscripts; for more details, the most recent base model and costing methods are available in Arredondo et al.^5^

## Results

Diabetes cases in older adults during the study period show constant increasing trends in the population of Mexico. These trends are stronger in the case of the insured population, showing an increase during the 2020–2022 period (2 887 207 [confidence interval {CI} 2 924 353 to 2 976 667] vs an increase of 3 176 483 [CI 3 324 550 to 3 383 120]). For total cases the increase was 4 441 857 (CI 4 512 270 to 4 571 793) vs 4 886 898 (CI 4 854 269 to 4 921 793)] (p<0.001).

For reasons of space and because 2020 is considered the year of greatest challenges for COVID-19 in terms of universal coverage for elderly patients with diabetes, 2020 was taken as the cut-off year to highlight the main results in the face of COVID-19 and the absence of a vaccine (highlighting that the model considers that by 2021 we have a vaccine). Table 1 shows the distribution of costs among the main items of economic impact in the management of diabetes in 2020. Direct costs represent 44% (case management and main complications) and indirect costs represent 56% (disability and premature mortality). With respect to the direct costs, it is worth noting that the greatest impact refers to medicines. Regarding the economic burden for the main complications, as well as for disability and premature mortality, we highlight that 2020 was the year with the greatest increase.

**Table 1.** Direct, indirect and total costs for healthcare attributable to older adults with diabetes to 2020 in Mexico (in US$^a^)

	Healthcare service provider					
Cost item	SSA	IMSS	ISSSTE	User's pocket	PHI	Total
Direct costs						
Consultations/diagnosis	57 877 932	130 645 783	30 566 984	253 171 470	14 606 041	486 868 210
Drugs	128 887 235	291 380 899	68 069 061	564 300 761	32 555 803	1 085 193 759
Hospitalisation	38 696 093	87 347 321	20 436 524	169 265 702	9 765 314	325 510 954
Retinopathy	11 767 729	26 562 894	6 214 875	37 436 227	2 159 782	84 141 508
Cardiovascular disease	10 697 960	24 148 083	539 495	65 513 373	3 779 620	104 678 530
Nephropathy	78 094 960	176 281 007	41 244 186	350 964 525	20 247 940	666 832 618
Neuropathy	3 851 258	8 693 307	2 033 957	7 487 244	431 957	22 497 723
Peripheral vascular disease	2 567 502	5 795 537	1 355 969	6 551 345	377 966	16 648 319
Total direct	332 440 670	750 854 833	170 461 049	1 454 690 644	83 924 421	2 792 371 617
Indirect costs						
Premature mortality	18 482 367	43 415 528	9 919 787	88 120 674	NA	159 938 356
Permanent disability	384 613 219	903 465 473	206 428 146	1 840 743 063	NA	3 335 249 901
** **Temporary disability	5 806 409	1 363 938	3 116 385	2 937 358	NA	13 224 088
Total indirect	408 901 994	948 244 941	219 464 317	1 931 801 093	NA	3 508 412 344
Total costs	741 342 664	1 699 099 774	389 925 366	3 386 491 737	83 924 421	6 300 783 961

Source: Arredondo et al. Costos y consecuencias financieras del cambio en el perfil epidemiológico en México. University of Montreal–INSP, Update of probabilistic models, June 2020.

^a^Exchange rate, June 2020: US$1 = 22.80 pesos.

IMSS, Mexican Institute for Social Security; ISSSTE, Institute for Social Security and Services for State Workers; NA, not applicable; PHI, private health insurance; SSA, Ministry of Health.

## Discussion

In the framework of effective universal coverage, the results of the update on recent trends in epidemiological and economic indicators of diabetes in older adults are of great relevance in terms of planning, financing and resource allocation for health intervention in diabetes.^5^ In the context of the COVID-19 pandemic, having evidence of recent trends by type of health provider will allow accurate decisions to be made for healthcare systems.^9^ Both the cost of case management and the epidemiological trends in cases of diabetes are similar to other findings published for Mexico and other countries.^8^ Indeed, the epidemiological and economic burden, as well as the out-of-pocket expenses, are similar to those of the latest Organisation for Economic Co-operation and Development reports and other studies,^10^ where the high economic burden represented by out-of-pocket expenses for healthcare is highlighted in countries such as Mexico, Chile and Brazil.

Regarding the limitations of the reported results, the costs of waiting times and transportation were not considered, because the original project was only limited to estimating the direct costs of care and indirect costs attributable to loss of productivity.

To the challenges of universal coverage from the epidemiological and economic burden for diabetes in older adults we must also add the challenges posed by the COVID-19 pandemic. Indeed, it is difficult enough to face the usual challenges for universal coverage of diabetes in older adults, but add to that the effects of diabetes–COVID comorbidity and it becomes especially difficult.^9^ Furthermore, conversion of the health system to deal with COVID-19 has stopped universal coverage programs that usually care for patients with diabetes as part of the national strategy against diabetes, obesity and hypertension.^4^ In this sense, it is extremely relevant to consider the following:

- It is important to highlight that because of the COVID-19 pandemic, the effectiveness, quality and efficiency, as well as the providing of health services under the universal coverage schemes, will be reduced for the entire population.^9^ There is evidence that even before the pandemic, 65–80% of users of health services with diabetes did not have the support of their medical unit to guarantee laboratory studies, required services and free delivery of pharmacological treatment.^11^
- From a population perspective, the COVID-19 pandemic is having effects on the behaviour of the general population, and patients with diabetes in particular.^12^ Special mention should be made of the effectiveness in complying with self-care and adherence to treatment that patients routinely carry out and that are highly recommended by the American Diabetes Association.^13^ Among such self-care actions it is necessary to highlight the effect of the pandemic on the behaviour of patients for glycaemic control, foot exams, ophthalmological exams, lipid control, annual influenza vaccine, blood pressure monitoring and dental exams. From the perspective of the health system, the usual practices for monitoring and control of diabetes are unfavourably affected by the changes implemented by health institutions in the face of the COVID-19 pandemic.^14^ In all Latin American countries, the main healthcare centres have been converted almost entirely to exclusively serve COVID-19 patients (including those with comorbid diabetes [average of 38–45% in some countries]).^14^^,^^15^
- The economic and social burden attributable to the effects of the COVID-19 pandemic in Mexico,^16^ particularly its possible effects on the trends reported here for 2020 in elderly patients with diabetes, should be emphasized. Indeed, the increase in indirect costs (due to temporary disability, permanent disability and premature death attributable to diabetes), mainly for the period 2020–2021, will substantially complicate the burden that it represents for health systems and for society as a whole.

## Data Availability

Data referring to the manuscript are available at the following institutional link: https://insp.mx/lineas-de-investigacion.html.
